# 3-(6-Bromo-4-oxo-4*H*-chromen-3-yl)-3,4-dihydro-2*H*-1,2,4-benzothia­diazine-1,1-dione

**DOI:** 10.1107/S1600536810044648

**Published:** 2010-11-06

**Authors:** Mariya al-Rashida, Saeed Ahmad Nagra, Islam Ullah Khan, George Kostakis, Ghulam Abbas

**Affiliations:** a Institute of Chemistry, University of the Punjab, Lahore, Pakistan; b Department of Chemistry, Government College University, Lahore, Pakistan; c Institute of Inorganic Chemistry, Karlsruhe Institute of Technology, D-76133 Karlsruhe, Germany

## Abstract

The mol­ecular structure of the title compound, C_16_H_11_BrN_2_O_4_S, is very similar to that of the previously reported fluoro analogue [al-Rashida *et al.* (2010 ▶). *Acta Cryst.* E**66**, o2707]. The mean planes of the bicyclic chromone system and the benzene ring of the benzothia­diazine derivative make a dihedral angle of 58.23 (8)°. An intra­molecular N—H⋯O hydrogen bond occurs. In the crystal, mol­ecules are linked into layers by N—H⋯O and C—H⋯O hydrogen bonds, generating an infinite two-dimensional network.

## Related literature

For background to the importance of the 1,2,4-benzothia­diazine-1,1-dioxide ring system in pharmaceutical and medicinal chemistry, see: Zhu *et al.* (2005 ▶); Kamal *et al.* (2007*a* ▶). For a survey on the anti­microbial activity of benzothia­diazine derivatives, see: Di Bella *et al.* (1983) ▶; Kamal *et al.* (2007*a* ▶,*b* ▶). The sulfonamide group is an active pharmacophore, see: Weisman & Brown (1964) ▶. For related structures, see: al-Rashida *et al.* (2009 ▶, 2010 ▶).
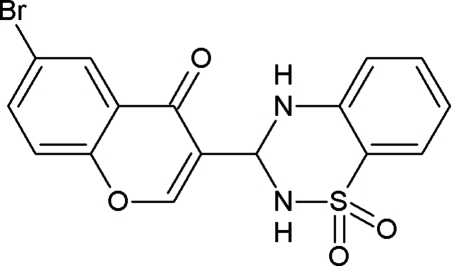


## Experimental

### 

#### Crystal data

C_16_H_11_BrN_2_O_4_S
*M* *_r_* = 407.24
Monoclinic, 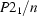

*a* = 7.0778 (4) Å
*b* = 8.6070 (6) Å
*c* = 25.6290 (16) Å
β = 94.607 (3)°
*V* = 1556.24 (17) Å^3^
*Z* = 4
Mo *K*α radiation
μ = 2.80 mm^−1^
*T* = 296 K
0.28 × 0.28 × 0.22 mm

#### Data collection

Bruker APEXII CCD area-detector diffractometer
Absorption correction: multi-scan (*SADABS*; Bruker, 2007 ▶) *T*_min_ = 0.475, *T*_max_ = 0.540
17309 measured reflections
3873 independent reflections
1969 reflections with *I* > 2σ(*I*)
*R*_int_ = 0.058

#### Refinement

*R*[*F* ^2^ > 2σ(*F* ^2^)] = 0.046
*wR*(*F* ^2^) = 0.103
*S* = 0.98
3873 reflections
223 parameters
2 restraints
H atoms treated by a mixture of independent and constrained refinement
Δρ_max_ = 0.50 e Å^−3^
Δρ_min_ = −0.51 e Å^−3^

### 

Data collection: *APEX2* (Bruker, 2007 ▶); cell refinement: *SAINT* (Bruker, 2007 ▶); data reduction: *SAINT*; program(s) used to solve structure: *SHELXS97* (Sheldrick, 2008 ▶); program(s) used to refine structure: *SHELXL97* (Sheldrick, 2008 ▶); molecular graphics: *ORTEP-3 for Windows* (Farrugia, 1997 ▶) and *Mercury* (Macrae *et al.*, 2006 ▶); software used to prepare material for publication: *publCIF* (Westrip, 2010 ▶).

## Supplementary Material

Crystal structure: contains datablocks I, global. DOI: 10.1107/S1600536810044648/zq2069sup1.cif

Structure factors: contains datablocks I. DOI: 10.1107/S1600536810044648/zq2069Isup2.hkl

Additional supplementary materials:  crystallographic information; 3D view; checkCIF report

## Figures and Tables

**Table 1:** Hydrogen-bond geometry (Å, °)

*D*—H⋯*A*	*D*—H	H⋯*A*	*D*⋯*A*	*D*—H⋯*A*
N4—H4*A*⋯O4	0.79 (3)	2.59 (3)	2.987 (3)	113 (3)
N4—H4*A*⋯O3^i^	0.79 (3)	2.23 (3)	2.999 (3)	164 (3)
C13—H13⋯O3^i^	0.93	2.54 (1)	3.314 (4)	141 (1)
N2—H2*A*⋯O2	0.84 (2)	2.67 (3)	3.222 (4)	124 (2)
C2—H2⋯O2	0.93	2.41 (1)	3.330 (4)	169 (1)
N2—H2*A*⋯O4	0.84 (2)	2.12 (3)	2.903 (4)	154 (3)

Symmetry code: (i) 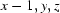.

**Fig. 1.**
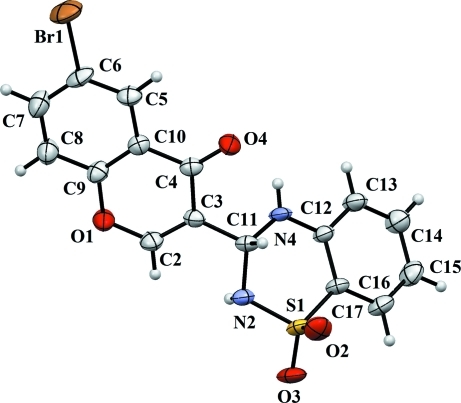
The molecular structure of the title compound with the atom-numbering scheme. Displacement ellipsoids are drawn at the 50% probability level.

**Fig. 2.**
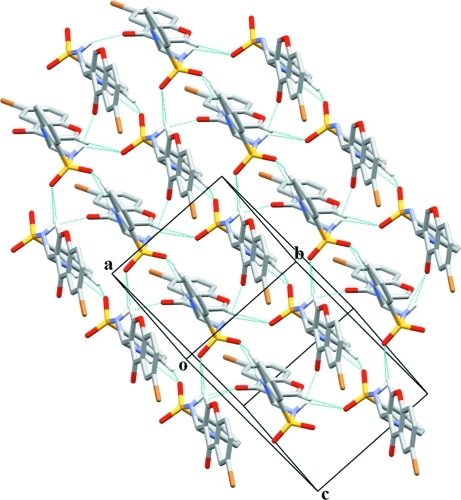
A packing diagram of the title compound showing hydrogen bonds as dashed lines.

## supplementary crystallographic information

### Comment

The 1,2,4-benzothiadiazine-1,1-dioxide ring system is of considerable importance in medicinal and pharmaceutical chemistry (Zhu *et al.*, 2005; Kamal *et al.*, 2007*a*). Novel products from reactions of 4- and 2-aminobenzenesulfonamide with 6-(un)substituted-4-oxo-4*H*-1-benzopyran-3-carboxaldehyde have already been reported by us (Mariya-al-Rashida *et al.*, 2009, 2010). In continuation of our project, the crystal structure of the title compound is reported here (Fig. 1).

In the crystal structure, the two rings of the chromone system (Br1, O1, O4, C2—C10) are coplanar making a dihedral angle of 1.0 (2)°. The carbon atom C11 deviates from the mean plane of the chromone ring by 0.016 (4) Å. The phenyl ring (C12—C17) of the benzothiadiazine moiety and the atoms S1, N4 and C11 are almost planar as well (rms deviation = 0.007) and make a dihedral angle of 58.23 (8)° with the mean plane of the bicyclic chromone system. The crystal structure is stabilized by intra- and intermolecular N—H···O and C—H···O hydrogen bonds which link the molecules into an infinite two-dimensional network (Fig. 2).

### Experimental

A solution of 2-aminobenzenesulfonamide (1.0 mmol) in 10 ml e thanol was slowly added to the stirred solution of 6-bromo-4-oxo-4*H*-1-benzopyran-3-carboxaldehyde (1.0 mmol) containing catalytic amount of *p*-toluene sulfonic acid (*p*-TsOH) and refluxed for 3 hrs. The resulting product was isolated by filtration, washed with ethanol, dried and recrystallized from hot ethanol and acetone (1:1) (yield 77%, m.p. 496 K).

### Refinement

The H atoms attached to N were located in a difference Fourier map and their coordinates were refined, with *U*_iso_(H) = 1.2*U*_eq_(N). The remaining H atoms were positioned geometrically with C-H = 0.93 and 0.98 Å for aromatic and methine H atoms, respectively, and constrained to ride on their parent atoms, with *U*_iso_(H) = 1.2*U*_eq_(C).

### Crystal data

**Table:**

C_16_H_11_BrN_2_O_4_S	*F*(000) = 816
*M**_r_* = 407.24	*D*_x_ = 1.738 Mg m^−^^3^
Monoclinic, *P*2_1_/*n*	Mo *K*α radiation, λ = 0.71073 Å
Hall symbol: -P 2yn	Cell parameters from 2932 reflections
*a* = 7.0778 (4) Å	θ = 3.1–22.1°
*b* = 8.6070 (6) Å	µ = 2.80 mm^−^^1^
*c* = 25.6290 (16) Å	*T* = 296 K
β = 94.607 (3)°	Needle, white
*V* = 1556.24 (17) Å^3^	0.28 × 0.28 × 0.22 mm
*Z* = 4	

### Data collection

**Table:**

Bruker APEXII CCD area-detector diffractometer	3873 independent reflections
Radiation source: fine-focus sealed tube	1969 reflections with *I* > 2σ(*I*)
graphite	*R*_int_ = 0.058
phi and ω scans	θ_max_ = 28.4°, θ_min_ = 1.6°
Absorption correction: multi-scan (*SADABS*; Bruker, 2007)	*h* = −9→9
*T*_min_ = 0.475, *T*_max_ = 0.540	*k* = −11→8
17309 measured reflections	*l* = −34→34

### Refinement

**Table:**

Refinement on *F*^2^	Primary atom site location: structure-invariant direct methods
Least-squares matrix: full	Secondary atom site location: difference Fourier map
*R*[*F*^2^ > 2σ(*F*^2^)] = 0.046	Hydrogen site location: inferred from neighbouring sites
*wR*(*F*^2^) = 0.103	H atoms treated by a mixture of independent and constrained refinement
*S* = 0.98	*w* = 1/[σ^2^(*F*_o_^2^) + (0.0423*P*)^2^ + 0.1442*P*] where *P* = (*F*_o_^2^ + 2*F*_c_^2^)/3
3873 reflections	(Δ/σ)_max_ < 0.001
223 parameters	Δρ_max_ = 0.50 e Å^−^^3^
2 restraints	Δρ_min_ = −0.51 e Å^−^^3^

### Special details

**Table:**

Geometry. All e.s.d.'s (except the e.s.d. in the dihedral angle between two l.s. planes) are estimated using the full covariance matrix. The cell e.s.d.'s are taken into account individually in the estimation of e.s.d.'s in distances, angles and torsion angles; correlations between e.s.d.'s in cell parameters are only used when they are defined by crystal symmetry. An approximate (isotropic) treatment of cell e.s.d.'s is used for estimating e.s.d.'s involving l.s. planes.
Refinement. Refinement of *F*^2^ against ALL reflections. The weighted *R*-factor *wR* and goodness of fit *S* are based on *F*^2^, conventional *R*-factors *R* are based on *F*, with *F* set to zero for negative *F*^2^. The threshold expression of *F*^2^ > σ(*F*^2^) is used only for calculating *R*-factors(gt) *etc*. and is not relevant to the choice of reflections for refinement. *R*-factors based on *F*^2^ are statistically about twice as large as those based on *F*, and *R*- factors based on ALL data will be even larger.

### Fractional atomic coordinates and isotropic or equivalent isotropic displacement parameters (Å^2^)

**Table:**

	*x*	*y*	*z*	*U*_iso_*/*U*_eq_	
S1	1.22103 (10)	0.73566 (10)	0.31055 (3)	0.0337 (2)	
O2	1.2394 (3)	0.5717 (3)	0.30708 (9)	0.0463 (6)	
O3	1.3885 (3)	0.8294 (3)	0.31425 (9)	0.0465 (6)	
N2	1.0884 (3)	0.7953 (3)	0.25943 (10)	0.0287 (6)	
H2A	1.085 (4)	0.893 (3)	0.2592 (12)	0.034*	
N4	0.8018 (3)	0.7790 (4)	0.30189 (10)	0.0371 (7)	
H4A	0.690 (4)	0.780 (4)	0.3005 (13)	0.045*	
Br1	−0.00741 (5)	0.67254 (5)	0.060030 (16)	0.06277 (19)	
C5	0.3194 (4)	0.7029 (4)	0.13029 (13)	0.0350 (8)	
H5	0.2622	0.6278	0.1498	0.042*	
C6	0.2305 (5)	0.7577 (4)	0.08503 (13)	0.0403 (9)	
C7	0.3086 (5)	0.8725 (4)	0.05590 (14)	0.0455 (9)	
H7	0.2429	0.9099	0.0256	0.055*	
C8	0.4824 (5)	0.9304 (4)	0.07182 (13)	0.0424 (9)	
H8	0.5374	1.0068	0.0523	0.051*	
C9	0.5766 (4)	0.8743 (4)	0.11749 (12)	0.0324 (8)	
O1	0.7509 (3)	0.9378 (3)	0.13110 (8)	0.0398 (6)	
C10	0.4984 (4)	0.7614 (4)	0.14695 (12)	0.0287 (7)	
C4	0.6000 (4)	0.7086 (4)	0.19593 (12)	0.0286 (8)	
O4	0.5369 (3)	0.6116 (3)	0.22456 (9)	0.0395 (6)	
C3	0.7845 (4)	0.7819 (4)	0.20758 (12)	0.0272 (7)	
C2	0.8469 (4)	0.8871 (4)	0.17524 (12)	0.0347 (8)	
H2	0.9665	0.9291	0.1838	0.042*	
C11	0.8977 (4)	0.7310 (4)	0.25680 (12)	0.0289 (7)	
H11	0.9066	0.6174	0.2568	0.035*	
C12	0.8888 (4)	0.7966 (4)	0.35104 (12)	0.0295 (8)	
C13	0.7836 (4)	0.8366 (4)	0.39290 (13)	0.0372 (8)	
H13	0.6531	0.8494	0.3871	0.045*	
C14	0.8682 (5)	0.8573 (4)	0.44190 (14)	0.0431 (9)	
H14	0.7942	0.8825	0.4691	0.052*	
C16	1.1691 (5)	0.8056 (4)	0.41215 (13)	0.0402 (9)	
H16	1.2999	0.7964	0.4183	0.048*	
C15	1.0631 (5)	0.8417 (4)	0.45229 (14)	0.0445 (9)	
H15	1.1196	0.8557	0.4860	0.053*	
C17	1.0843 (4)	0.7823 (4)	0.36172 (12)	0.0291 (8)	

### Atomic displacement parameters (Å^2^)

**Table:**

	*U*^11^	*U*^22^	*U*^33^	*U*^12^	*U*^13^	*U*^23^
S1	0.0158 (4)	0.0421 (6)	0.0427 (5)	0.0059 (4)	−0.0002 (4)	−0.0004 (4)
O2	0.0393 (14)	0.0381 (15)	0.0622 (17)	0.0148 (11)	0.0081 (12)	0.0055 (12)
O3	0.0160 (11)	0.0624 (17)	0.0608 (16)	−0.0034 (11)	0.0001 (11)	−0.0038 (13)
N2	0.0172 (13)	0.0291 (15)	0.0393 (16)	−0.0005 (12)	0.0005 (12)	0.0016 (14)
N4	0.0121 (12)	0.070 (2)	0.0293 (16)	0.0004 (14)	0.0002 (13)	−0.0041 (14)
Br1	0.0358 (2)	0.0836 (4)	0.0653 (3)	−0.0100 (2)	−0.01830 (19)	−0.0107 (2)
C5	0.0287 (17)	0.035 (2)	0.040 (2)	−0.0072 (15)	−0.0010 (16)	−0.0056 (17)
C6	0.0290 (19)	0.049 (2)	0.040 (2)	0.0009 (17)	−0.0140 (16)	−0.0148 (18)
C7	0.051 (2)	0.046 (2)	0.037 (2)	−0.0006 (19)	−0.0116 (18)	0.0050 (18)
C8	0.047 (2)	0.042 (2)	0.036 (2)	−0.0048 (18)	−0.0029 (18)	0.0077 (17)
C9	0.0332 (18)	0.032 (2)	0.0313 (19)	−0.0038 (15)	−0.0044 (15)	−0.0040 (16)
O1	0.0367 (13)	0.0447 (15)	0.0367 (14)	−0.0182 (11)	−0.0056 (11)	0.0071 (11)
C10	0.0283 (17)	0.0265 (19)	0.0309 (18)	−0.0002 (14)	0.0009 (15)	−0.0027 (15)
C4	0.0250 (17)	0.0269 (19)	0.0338 (19)	−0.0007 (14)	0.0014 (15)	−0.0058 (16)
O4	0.0347 (13)	0.0426 (14)	0.0406 (14)	−0.0153 (11)	−0.0015 (11)	0.0104 (12)
C3	0.0219 (16)	0.0315 (19)	0.0286 (17)	−0.0038 (14)	0.0034 (14)	−0.0041 (15)
C2	0.0283 (18)	0.040 (2)	0.035 (2)	−0.0090 (16)	−0.0022 (16)	−0.0028 (17)
C11	0.0171 (15)	0.0334 (19)	0.0361 (19)	−0.0019 (14)	0.0027 (14)	−0.0035 (15)
C12	0.0190 (15)	0.038 (2)	0.0303 (18)	0.0018 (14)	−0.0024 (14)	−0.0023 (15)
C13	0.0213 (16)	0.052 (2)	0.038 (2)	0.0039 (16)	0.0010 (15)	−0.0052 (18)
C14	0.039 (2)	0.051 (2)	0.039 (2)	0.0055 (17)	0.0017 (17)	−0.0085 (18)
C16	0.0257 (18)	0.048 (2)	0.044 (2)	0.0029 (16)	−0.0094 (17)	−0.0032 (18)
C15	0.044 (2)	0.054 (3)	0.033 (2)	0.0006 (18)	−0.0092 (17)	−0.0068 (18)
C17	0.0167 (15)	0.0338 (19)	0.0363 (19)	0.0029 (13)	−0.0010 (14)	−0.0017 (15)

### Geometric parameters (Å, °)

**Table:**

S1—O2	1.421 (2)	C9—C10	1.375 (4)
S1—O3	1.431 (2)	O1—C2	1.345 (4)
S1—N2	1.632 (3)	C10—C4	1.468 (4)
S1—C17	1.738 (3)	C4—O4	1.219 (3)
N2—C11	1.456 (4)	C4—C3	1.459 (4)
N2—H2A	0.84 (2)	C3—C2	1.327 (4)
N4—C12	1.365 (4)	C3—C11	1.504 (4)
N4—C11	1.446 (4)	C2—H2	0.9300
N4—H4A	0.79 (3)	C11—H11	0.9800
Br1—C6	1.900 (3)	C12—C13	1.398 (4)
C5—C6	1.359 (4)	C12—C17	1.394 (4)
C5—C10	1.398 (4)	C13—C14	1.359 (4)
C5—H5	0.9300	C13—H13	0.9300
C6—C7	1.380 (5)	C14—C15	1.391 (5)
C7—C8	1.359 (5)	C14—H14	0.9300
C7—H7	0.9300	C16—C15	1.357 (5)
C8—C9	1.387 (4)	C16—C17	1.395 (4)
C8—H8	0.9300	C16—H16	0.9300
C9—O1	1.368 (4)	C15—H15	0.9300
			
O2—S1—O3	119.01 (14)	O4—C4—C10	123.3 (3)
O2—S1—N2	108.16 (14)	C3—C4—C10	114.2 (3)
O3—S1—N2	107.20 (14)	C2—C3—C4	120.3 (3)
O2—S1—C17	109.63 (15)	C2—C3—C11	122.8 (3)
O3—S1—C17	109.16 (14)	C4—C3—C11	117.0 (3)
N2—S1—C17	102.36 (14)	C3—C2—O1	125.2 (3)
C11—N2—S1	112.9 (2)	C3—C2—H2	117.4
C11—N2—H2A	111 (2)	O1—C2—H2	117.4
S1—N2—H2A	109 (2)	N4—C11—N2	110.3 (2)
C12—N4—C11	124.3 (2)	N4—C11—C3	109.6 (2)
C12—N4—H4A	115 (3)	N2—C11—C3	111.0 (3)
C11—N4—H4A	120 (3)	N4—C11—H11	108.6
C6—C5—C10	118.8 (3)	N2—C11—H11	108.6
C6—C5—H5	120.6	C3—C11—H11	108.6
C10—C5—H5	120.6	N4—C12—C13	120.5 (3)
C5—C6—C7	122.2 (3)	N4—C12—C17	122.6 (3)
C5—C6—Br1	119.4 (3)	C13—C12—C17	116.9 (3)
C7—C6—Br1	118.5 (2)	C14—C13—C12	121.3 (3)
C8—C7—C6	119.5 (3)	C14—C13—H13	119.4
C8—C7—H7	120.3	C12—C13—H13	119.4
C6—C7—H7	120.3	C13—C14—C15	121.4 (3)
C7—C8—C9	119.2 (3)	C13—C14—H14	119.3
C7—C8—H8	120.4	C15—C14—H14	119.3
C9—C8—H8	120.4	C15—C16—C17	120.8 (3)
O1—C9—C10	122.5 (3)	C15—C16—H16	119.6
O1—C9—C8	116.0 (3)	C17—C16—H16	119.6
C10—C9—C8	121.5 (3)	C16—C15—C14	118.5 (3)
C2—O1—C9	118.0 (2)	C16—C15—H15	120.7
C9—C10—C5	118.8 (3)	C14—C15—H15	120.7
C9—C10—C4	119.8 (3)	C16—C17—C12	121.0 (3)
C5—C10—C4	121.3 (3)	C16—C17—S1	120.5 (2)
O4—C4—C3	122.5 (3)	C12—C17—S1	118.4 (2)
			
O2—S1—N2—C11	61.9 (2)	C9—O1—C2—C3	−1.5 (5)
O3—S1—N2—C11	−168.6 (2)	C12—N4—C11—N2	−35.8 (4)
C17—S1—N2—C11	−53.8 (2)	C12—N4—C11—C3	−158.3 (3)
C10—C5—C6—C7	−2.0 (5)	S1—N2—C11—N4	61.6 (3)
C10—C5—C6—Br1	177.0 (2)	S1—N2—C11—C3	−176.8 (2)
C5—C6—C7—C8	1.9 (5)	C2—C3—C11—N4	114.2 (3)
Br1—C6—C7—C8	−177.2 (3)	C4—C3—C11—N4	−66.5 (3)
C6—C7—C8—C9	−0.9 (5)	C2—C3—C11—N2	−7.9 (4)
C7—C8—C9—O1	179.9 (3)	C4—C3—C11—N2	171.4 (2)
C7—C8—C9—C10	0.1 (5)	C11—N4—C12—C13	−177.1 (3)
C10—C9—O1—C2	−0.4 (4)	C11—N4—C12—C17	5.6 (5)
C8—C9—O1—C2	179.7 (3)	N4—C12—C13—C14	−178.8 (3)
O1—C9—C10—C5	179.9 (3)	C17—C12—C13—C14	−1.4 (5)
C8—C9—C10—C5	−0.2 (5)	C12—C13—C14—C15	0.9 (5)
O1—C9—C10—C4	2.1 (5)	C17—C16—C15—C14	−1.0 (5)
C8—C9—C10—C4	−178.0 (3)	C13—C14—C15—C16	0.3 (5)
C6—C5—C10—C9	1.2 (5)	C15—C16—C17—C12	0.5 (5)
C6—C5—C10—C4	179.0 (3)	C15—C16—C17—S1	−179.9 (3)
C9—C10—C4—O4	178.2 (3)	N4—C12—C17—C16	178.0 (3)
C5—C10—C4—O4	0.5 (5)	C13—C12—C17—C16	0.6 (5)
C9—C10—C4—C3	−2.0 (4)	N4—C12—C17—S1	−1.6 (4)
C5—C10—C4—C3	−179.7 (3)	C13—C12—C17—S1	−179.0 (2)
O4—C4—C3—C2	−179.9 (3)	O2—S1—C17—C16	89.7 (3)
C10—C4—C3—C2	0.3 (4)	O3—S1—C17—C16	−42.3 (3)
O4—C4—C3—C11	0.8 (4)	N2—S1—C17—C16	−155.7 (3)
C10—C4—C3—C11	−179.0 (3)	O2—S1—C17—C12	−90.7 (3)
C4—C3—C2—O1	1.4 (5)	O3—S1—C17—C12	137.3 (3)
C11—C3—C2—O1	−179.3 (3)	N2—S1—C17—C12	24.0 (3)

### Hydrogen-bond geometry (Å, °)

**Table:**

*D*—H···*A*	*D*—H	H···*A*	*D*···*A*	*D*—H···*A*
N4—H4A···O4	0.79 (3)	2.59 (3)	2.987 (3)	113 (3)
N4—H4A···O3^i^	0.79 (3)	2.23 (3)	2.999 (3)	164 (3)
C13—H13···O3^i^	0.93	2.54 (1)	3.314 (4)	141 (1)
N2—H2A···O2	0.84 (2)	2.67 (3)	3.222 (4)	124 (2)
C2—H2···O2	0.93	2.41 (1)	3.330 (4)	169 (1)
N2—H2A···O4	0.84 (2)	2.12 (3)	2.903 (4)	154 (3)

Symmetry codes: (i) *x*−1, *y*, *z*.
